# Affect labeling: a promising new neuroscience-based approach to treating combat-related PTSD in veterans

**DOI:** 10.3389/fpsyg.2024.1270424

**Published:** 2024-06-07

**Authors:** Lisa J. Burklund, Carolyn D. Davies, Andrea Niles, Jared B. Torre, Lily Brown, Meghan Vinograd, Matthew D. Lieberman, Michelle G. Craske

**Affiliations:** ^1^Department of Psychology, University of California, Los Angeles, Los Angeles, CA, United States; ^2^NeuroGen Technologies Inc., Los Angeles, CA, United States

**Keywords:** posttraumatic stress disorder, veterans, affect labeling, amygdala, intervention, combat, inhibitory regulation, PTSD

## Abstract

**Introduction:**

A significant portion of individuals exposed to combat-related trauma will develop posttraumatic stress disorder (PTSD), a severe, debilitating disorder with adverse impacts on both mental and physical functioning. Current treatments are effective for many individuals, however, there is a need for new treatment approaches to improve outcomes in PTSD and address the many existing barriers to seeking or completing treatment.

**Methods:**

In this open trial pilot study, we tested a novel, brief, computer-based intervention for PTSD utilizing “affect labeling” that was inspired by recent advances in neuroscience with U.S. veterans.

**Results:**

As expected, pre-intervention clinical and fMRI neuroimaging data indicated that U.S. veterans with combat-related PTSD (*N* = 20) had significantly higher PTSD symptoms, depression symptoms, and amygdala reactivity to trauma cues than trauma-exposed healthy control veterans (*N* = 20). Veterans with PTSD who completed the affect labeling intervention (*N* = 13) evidenced reduced PTSD symptoms and these reductions were correlated with reductions in amygdala reactivity.

**Discussion:**

Results from this initial proof-of-concept study are intriguing and suggest that affect labeling training offers significant potential as a novel, cost-effective, computer-based intervention for PTSD. Implications and next steps for further developing affect labeling interventions for PTSD are discussed.

**Clinical Trial Registration:**

https://clinicaltrials.gov/, identifier NCT05924399.

## Introduction

A significant portion of persons exposed to combat-related trauma will develop posttraumatic stress disorder (PTSD), a severe, debilitating disorder with adverse impacts on both mental and physical functioning. For U.S. military service personnel in particular, it is estimated that 93% are exposed to at least one traumatic event during their active duty military service (Dedert et al., 2009) and approximately 14–22% develop PTSD (Tanielian and Jaycox, 2008; Seal et al., 2009). While existing evidence-based treatments such as prolonged exposure, cognitive processing therapy, and pharmacological approaches provide benefit for many individuals with PTSD, as many as 50% do not show clinically significant response rates (Loerinc et al., 2015). Furthermore, as few as 50% of veterans in need of treatment for PTSD receive minimally-adequate care (Hundt et al., 2014).

Common barriers to treatment seeking and adherence for veterans with PTSD include the stigma of PTSD (Hoge et al., 2004), negative perceptions regarding treatment availability and quality (Desai et al., 2005), and logistical issues such as scheduling difficulties and inadequate transportation. Additional issues include the lack of trained clinicians and costs associated individual-based therapies (Tanielian and Jaycox, 2008). Barriers associated with pharmacological treatments are equal if not greater and include resistance to medications, side effects, contraindications, and preference for psychological treatments (McHugh et al., 2013).

There is a need for new treatment approaches to improve outcomes in PTSD, particularly cost-effective treatment programs that are easily accessible and appealing for individuals who may be otherwise disinclined to pursue formal mental health services. In this proof-of-concept pilot study, we aimed to address many of the above-mentioned barriers by testing a novel, brief, computer-based cognitive intervention for PTSD utilizing “affect labeling” that was developed from and inspired by recent advances in neuroscience.

A core component of PTSD is an inability to effectively down-regulate negative emotions in response to trauma reminders. This phenomenon is theorized to involve increased reactivity of primitive neural regions that mediate threat response (e.g., the amygdala) as well as decreased efficacy of top-down neural regulatory control regions including the prefrontal cortex (PFC) that dampen such responses appropriately. In other words, PTSD can be characterized as involving both a hypersensitive danger “alarm system” as well as a dysfunctional system for shutting off the alarm when there is no real danger present. Neuroimaging research supports this theoretical framework in that it has shown that many individuals with PTSD exhibit heightened responsivity of the amygdala during trauma-related and other emotional processing as well as impaired down-regulation or ‘extinction’ of amygdala-based fear responses by the ventral medial prefrontal cortex (VMPFC; see Hayes et al., 2012 for a review). The amygdala is a bilateral structure of the more primitive limbic system involved in the acquisition and detection of learned fear responses such as those that characterize PTSD (LeDoux, 1996). The amygdala is theorized to be central to the detection of potential threat as it has been shown to activate in response to threatening stimuli in healthy individuals (Phelps, 2006) and to produce heightened responses in individuals with anxiety disorders, including PTSD (Etkin and Wager, 2007). The VMPFC is theorized to play a fundamental role in the extinction or suppression of fear-related memories (Morgan et al., 1993). As such, VMPFC regulation of fear responses appears to represent a key neural mechanism of exposure-based treatments, which ostensibly represent the *in-vivo* application of extinction learning.

However, recent research suggests that a distinct *lateral* PFC route, involving ventrolateral PFC (VLPFC) control over the amygdala, may also be compromised in PTSD. Much previous work in the fields of cognitive and affective neuroscience has identified the VLPFC, particularly in the right hemisphere (RVLPFC), as central to *inhibitory regulation* in healthy individuals (Cohen et al., 2013). Inhibitory regulation or inhibitory control refers to the disruption, suppression, or prevention of prepotent responses to maintain goal-directed behavior, and operates across multiple domains (e.g., emotional, cognitive, motor). One example of inhibitory regulation is affect labeling, which involves verbally labeling the emotional content of a stimulus or labeling feelings in response to a stimulus, and has been shown in numerous studies to consistently engage the RVLPFC to down-regulate, or inhibit, the amygdala in healthy individuals (e.g., Lieberman et al., 2007; Burklund et al., 2014; Torre and Lieberman, 2018). It is not particularly surprising that the simple process of affect labeling is effective in down-regulating amygdala/affective responses given that the process of affect labeling constitutes a mechanism of many forms of psychotherapy (e.g., talking about your feelings). Neuroimaging studies have likewise shown that motor inhibition, such as that seen in classic go-nogo studies wherein an individual inhibits a prepotent motor response on infrequent trials, also consistently involves the RVLPFC (Simmonds et al., 2008), and even down-regulates incidental amygdala activity in the process (Berkman et al., 2009). In separate clinical work, PTSD has been associated with impairments in inhibitory processing at the behavioral level (Falconer et al., 2008; Aupperle et al., 2011; DeGutis et al., 2015). No previous studies have directly and quantitatively examined possible neural impairments in emotional inhibitory regulation of the amygdala-mediated fear response by the RVLPFC in PTSD; however, related research supports this hypothesis by demonstrating abnormally diminished RVLPFC activity in a motor control inhibition task (Falconer et al., 2008) and when imagining one’s trauma (Hayes et al., 2012). Based on such research, it would then follow that correction of RVLPFC-based inhibitory impairment in PTSD may form the basis for a novel treatment target.

Synthesizing these previous findings, we set out to investigate whether individuals with PTSD exhibit improvements in PTSD symptoms, reflecting improved emotion regulation, following repeated practice with inhibitory regulation strategies designed to strengthen RVLPFC-based inhibitory capacity. In the current pilot study, we tested a novel affect labeling-based intervention for combat-related PTSD in veterans. Specifically, we tested whether veterans with combat-related PTSD exhibited improvements in PTSD symptoms following repeated practice with affect labeling and other forms of inhibitory regulation (e.g., motor inhibition), which would ostensibly strengthen RVLPFC-based inhibitory capacity. Veterans with PTSD and trauma-exposed healthy control veterans completed baseline assessments involving a clinical interview, questionnaires, and an fMRI scan. Those with PTSD then underwent a three-week affect labeling/inhibitory regulation intervention, followed by a post-training assessment similar to the baseline assessment to allow us to assess effects of the training. We predicted that participants with PTSD would exhibit amygdala hyperreactivity to trauma-related stimuli relative to healthy controls, replicating previous studies, and thereby effectively serving to as a manipulation check of the assumption of maladaptive threat-detection at the neural level. After completing the intervention, individuals with PTSD were predicted to exhibit decreased PTSD symptoms reflecting an enhanced capacity for inhibiting maladaptive emotional responses as well as corresponding decreased amygdala reactivity to trauma-related stimuli, reflecting concomitant underlying changes in neural functioning.

## Materials and methods

### Participants

Participants included 20 individuals who met DSM-5 criteria for PTSD or other Trauma-Related Disorder, as assessed using the Clinician-Administered PTSD Scale for DSM-5 (CAPS-5; Weathers et al., 2013a), the gold standard for clinical assessment of PTSD, which is a 30-item structured interview that yields a symptom severity score (“CAPS-5 scores”) based on 20 items that reflect DSM-5 PTSD symptoms using a 5-point Likert scale (0 = “absent” to 4 = “extreme/incapacitating”), and a clinical severity rating (CSR) ≥ 4 on a 0–8 scale assigned by the interviewer reflecting clinically significant distress or impairment (all described as “participants with PTSD” or “PTSD” in this manuscript). Twenty trauma-exposed healthy control participants (“TEHC”) were also included; they did not meet DSM-5 criteria for current/lifetime PTSD or any other psychiatric disorder, except mild substance use disorder and adjustment disorder. All study participants were U.S. military veterans with deployment experience, 18–45 years old, English-speaking, and right-handed (in order to allow comparison of neural activity across participants). Additionally, all participants met DSM-5 criterion “A” for PTSD, specifically involving exposure to a combat-related traumatic event related to their military service, although this may have taken a variety of forms (e.g., injury to self, witnessing death of another). Individuals were excluded for (1) standard fMRI contraindications including metallic implants or other non-removable metal in the body (e.g., shrapnel, surgical screws, etc.), claustrophobia, and pregnancy, (2) serious unstable medical conditions, (3) intellectual impairment, (4) bipolar disorder, psychosis, delusional disorder, and suicidality, (5) a history of moderate to severe traumatic brain injury, (6) moderate to severe substance use disorder within the last 6 months, (7) recent modifications to psychotropic medication status, (8) recently initiated psychotherapy (within the last 3 months), and (9) chronic or repeated neglect/maltreatment, sexual abuse, physical abuse, emotional abuse, or domestic violence prior to the age of 7, given evidence for adverse brain development and structural abnormalities in this subgroup of individuals. Participants were recruited via postings in the community, internet, and veteran organizations, and were compensated for their participation. The control group was recruited to be matched on military experience and exposure to a combat-related trauma only. One TEHC and 3 PTSD participants reported taking regular medication; however, we do not have data on the type of medication participants were taking.

### Procedure

**Figure 1:**
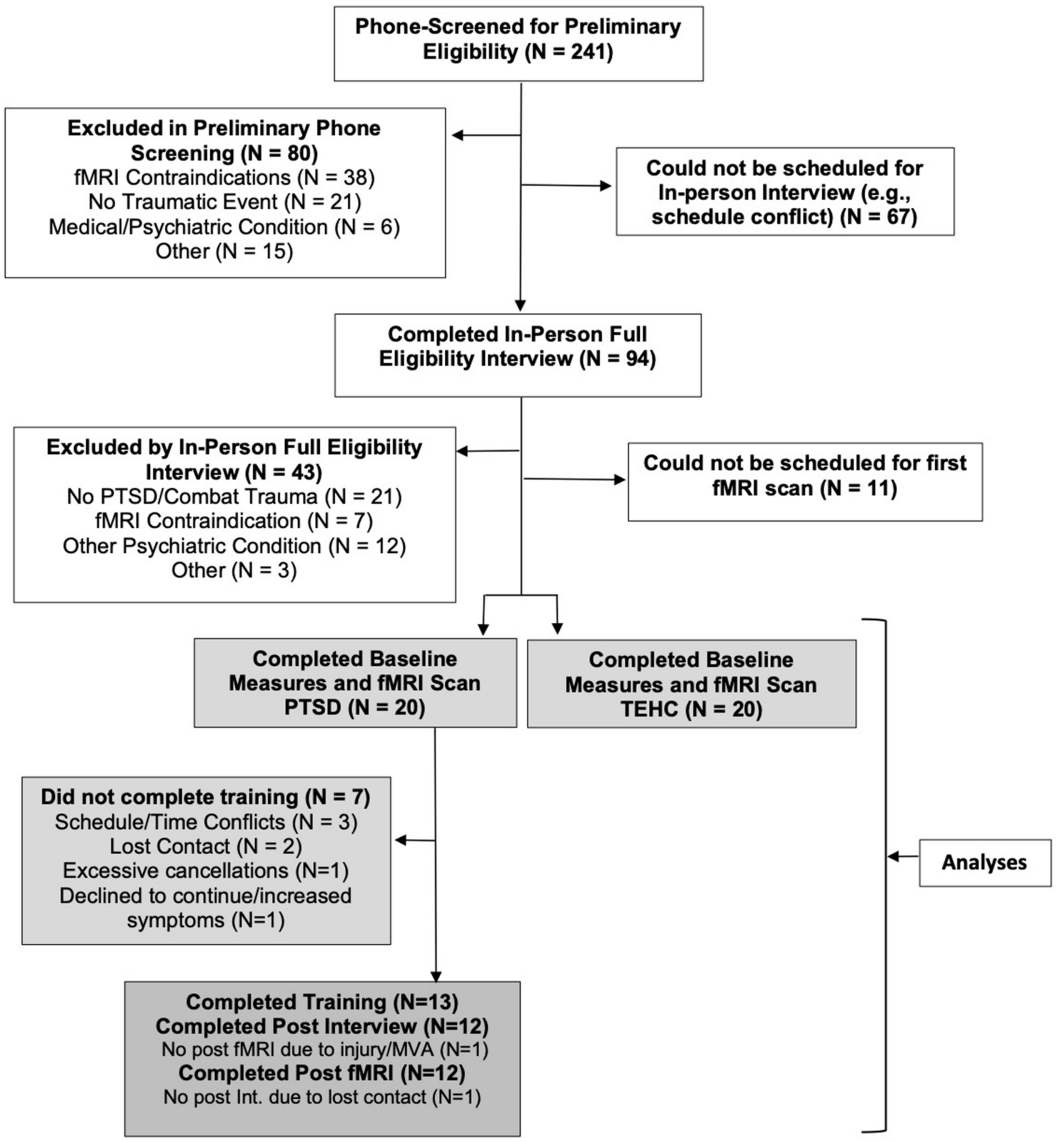
Participant flow diagram.

Following a brief telephone screening, potentially eligible participants provided informed consent and then completed an in-person diagnostic interview using the CAPS-5, SCID-5-Research Version (First et al., 2015), and Ohio State Traumatic Brain Injury Identification Method (Corrigan and Bogner, 2007) to determine eligibility and exclusion. Interviewers included graduate students and research staff who were trained and certified as reliable diagnosticians. Eligible participants completed self-report measures, described below, and underwent an fMRI scanning session during which they completed multiple tasks, including a combat picture task to assess neural responses to trauma-related stimuli. Participants with PTSD then completed a three-week affect labeling training intervention, described below. Approximately 1–2 weeks after the last training session, participants with PTSD completed a second fMRI session identical to the first, followed by a second clinical interview and questionnaires approximately 1 month after the second fMRI session, to assess changes in clinical status and symptoms. All in-person sessions were completed at UCLA. Participants were compensated $20 for each session except the final fMRI session, for which they were compensated $100, and the final clinical interview, for which they were compensated $10. This study was approved by the UCLA Institutional Review Board and registered with ClinicalTrials.gov (ID# NCT05924399). See Figure 1 for a participant flow diagram.

#### fMRI combat picture task

While in the fMRI scanner, participants passively viewed aversive combat-related images from the Military Affective Picture System (MAPS; Goodman et al., 2016; “Observe” condition) and completed a non-emotional shape-matching control condition (“Shape Match” condition) wherein they chose a shape that matched the target shape at the top of the screen, presented in a blocked design. Additional conditions and tasks were included, but are not discussed in this manuscript. Combat images consisted of genuine war photos, the majority taken in Iraq and Afghanistan; examples include photos of caskets, masked insurgents, and pictures of deceased and wounded soldiers and civilians, thereby constituting trauma-relevant stimuli. Conditions were presented in a blocked design, with five 5-s trials per block, and four blocks per condition. Each block was followed by a 12-s fixation cross presentation.

#### Affect labeling intervention

**Figure 2:**
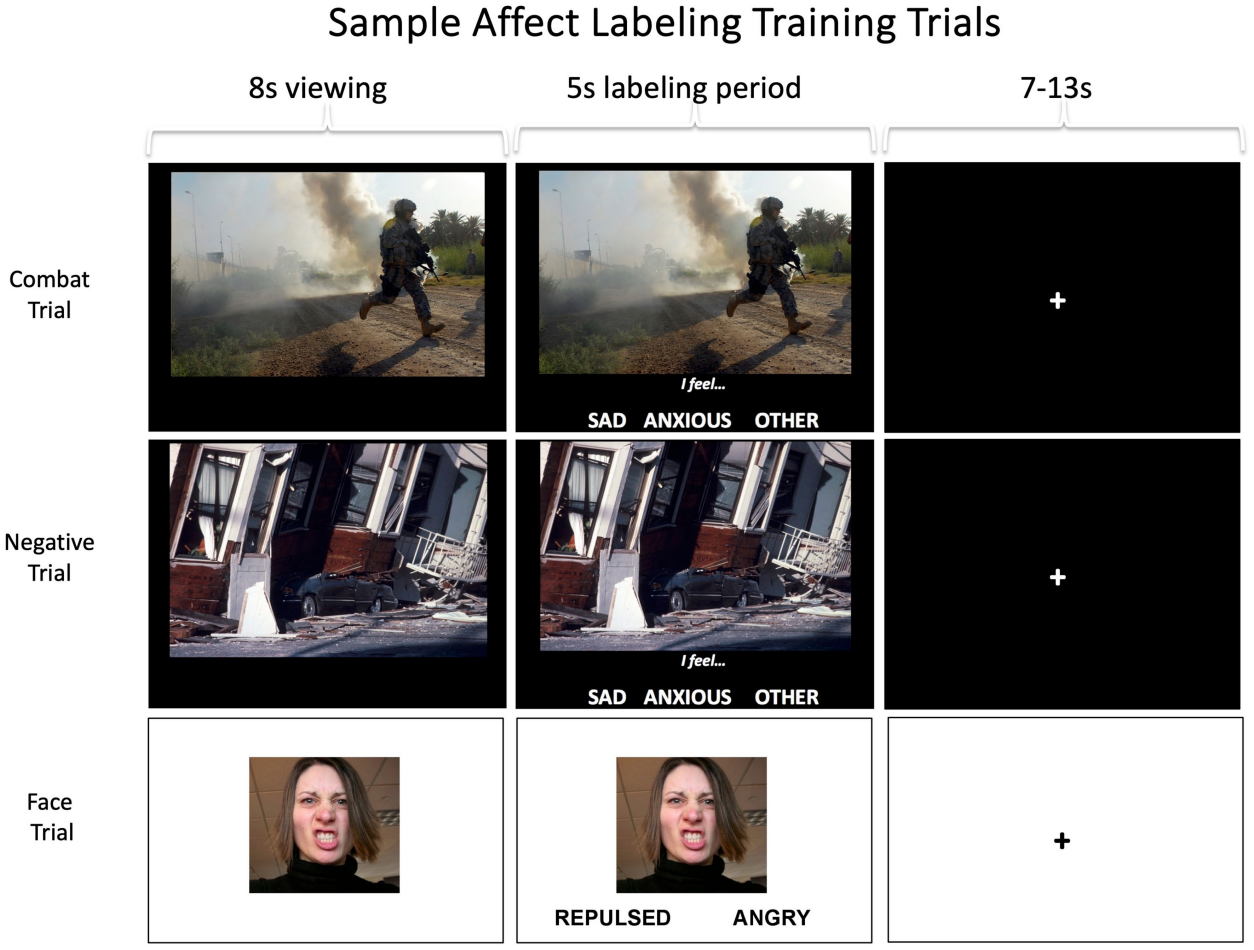
Sample screens from combat, negative, and facial expression affect labeling trials. Participants completed two five-minute blocks of each trial type, with 13 images per block and each image on screen for a total of 13 seconds. Participants also completed two five-minutes blocks of a standard go-nogo task. The images shown here are not actual stimuli but are similar. Top image: Credit to DVIDSHUB, reproduced with permission from Staff Sgt. Mark Burrell; https://creativecommons.org/licenses/by/2.0/. Middle image: Credit to USGS Denver Library Photographic Collection, reproduced with permission from USGS; public domain. Bottom image: Reproduced with permission from Lara Schneider; https://creativecommons.org/licenses/by-nc-nd/2.0/.

Participants with PTSD completed six one-hour sessions of the affect labeling training intervention, approximately twice a week for 3 weeks, although participants were allowed to make up missed sessions beyond 3 weeks as necessary. The training program was presented via computer, and participants responded via keyboard button presses. Training was conducted in a private room with only the participant and a research staff member present. Each 1-h session included 40 min of training, and 20 min of set up and other administrative tasks. During each session, participants completed eight five-minute blocks of inhibitory regulation training. This included two blocks each of four different types of inhibitory processing. In the first type, participants were shown 13 distinct aversive combat-related images from the MAPS set (similar to but distinct from those shown in the fMRI scanner) one at a time, and for each image, they chose one of three affect-related labels at the bottom of the screen that best described how they felt while viewing the image (e.g., I feel… angry, scared) or that described emotionally-evocative aspects of the image (e.g., I see… gun, wound; see Figure 2 for sample trials). The image was displayed by itself for 8 s, affective labels then appeared on-screen and the participant had 5 s to make their choice via button response. The trials were separated by 7–13 s of fixation crosshair, jittered to facilitate collection of psychophysiological responses, the results of which will be presented in a separate manuscript. The second and third types of inhibitory training involved a similar affect labeling format and procedure, but instead of trauma-relevant images, generally aversive images (IAPS; Lang et al., 2008) and negative emotional facial expressions (NimStim Face Stimulus Set; Tottenham et al., 2009) were presented for affect labeling. For the negative emotional faces, participants were asked to label the emotional expression of the target face rather than their own emotions. The final type of inhibitory training involved completion of a Go-NoGo motor inhibition task. As such, while the entire intervention involved inhibitory regulation training, 75% specifically utilized affective labeling, and only 25% was clearly trauma-relevant. However, we note that in the context of viewing aversive combat-related images, the negative scenes (e.g., a surgical amputation) and emotional facial expressions (e.g., an angry face) could be construed as trauma-relevant. We chose to include multiple types of inhibitory regulation in the intervention as generalized, elaborative practice facilitates learning; although we note that future work should tease apart the most potent aspects of the training.

### Measures

Participants completed the PTSD Checklist for DSM-5 (PCL-5; Weathers et al., 2013b) to provide an index of self-reported PTSD symptoms. The PCL-5 is a 20-item self-report measure that assesses the 20 DSM-5 symptoms of PTSD using a 5-point Likert scale of how much they were bothered by each symptom (0 = “not at all” to 4 = “extremely”). Scores are summed and higher scores represent greater self-reported PTSD symptoms. Participants completed the anhedonic depression subscale of the Mood and Anxiety Symptom Questionnaire – Mini Version (Mini-MASQ; Casillas and Clark, 2000) to provide an index of depression symptoms. The Mini-MASQ is a 26-item self-report measure that has participants rate how much they have experienced various experiences using a 5-point Likert scale (1 = “not at all” to 5 = “extremely”) and includes an 8-item anhedonic depression subscale specifically assessing loss of interest and pleasure as well as positive emotional experiences (reverse scored) with higher scores reflecting greater anhedonic depression. Participants completed the Combat Exposure Scale (CES; Keane et al., 1989) to provide a measure of combat exposure. The CES is a 7-item self-report measure that assesses wartime stressors experienced by military personnel (e.g., “under enemy fire”). Items are rated on a 5-point frequency scale (1 = “no” or “never” to 5 = “51+ times”), with higher scores reflecting greater exposure to combat. Participants completed the Positive and Negative Affective Schedules (PANAS; Watson et al., 1988) to provide measures of general affect. The PANAS is a 20-item measure that has participants rate how much various affective descriptors apply to them using a 5-point Likert scale (0 = “very slightly or not at all” to 5 = “extremely”) and can be split into positive and negative subscales with higher scores reflecting greater positive and negative affect, respectively. Participants also completed a five-item treatment expectancy questionnaire (Devilly and Borkovec, 2000) adapted for this study at the end of the second and final intervention sessions to assess how much they believed they would benefit from the intervention using a 9-point Likert scale (0 = “not at all” to 8 = “very”).

### Data analysis

#### fMRI data collection

Tasks were presented via the Psychophysics Toolbox (Brainard, 1997; Kleiner et al., 2007) in the MATLAB environment version 7.4. Participants viewed the task via MR-compatible LCD goggles while in-scanner responses were made via a button response box held in the subject’s right hand.

#### Image acquisition

Imaging data were acquired via a Siemens Tim Trio 3 tesla MRI scanner at the UCLA Staglin Center for Cognitive Neuroscience. We acquired functional T2*-weighted echo planar image volumes (EPIs; slice thickness = 4 mm, gap = 1 mm, 33 oblique axial slices, TR = 2000 ms, TE = 30 ms, flip angle = 75°, matrix = 64×64, FOV = 220 mm). Two structural scans were acquired including a matched bandwidth high-resolution T2-weighted echo-planar image (spin echo; slice thickness = 4 mm, no gap between slices, 34 slices, TR = 5,000 ms, TE = 34 ms, flip angle = 90°, matrix = 128 × 128, FOV = 196 mm) and a T1-weighted, magnetization prepared, rapid-acquisition, gradient echo anatomical scan (MPRAGE slice thickness = 1 mm, gap = 0.5 mm, 160 slices, TR = 1900 ms, TE = 3.43 ms, flip angle = 9°, matrix = 256 × 256, FOV = 256 mm) to facilitate image normalization.

#### Preprocessing

Imaging data were analyzed using SPM8 (Wellcome Department of Cognitive Neurology, Institute for Neurology, London, UK). All images were first manually reoriented to align brains along a horizontal AC-PC line with the image origin at the anterior commissure; structural images were reoriented independently but functional images were reoriented using parameters from the first run’s first image applied to each subsequent volume within the task. All functional images were then realigned to the mean volume within the appropriate run to correct for head motion. High resolution MPRAGE structural images were co-registered to a mean EPI using the T2-weighted echo planar structural as a mediating co-registration step. MPRAGE anatomical images were then normalized using the New Segmentation routine in SPM8 to warp them into Montreal Neurological Institute space (resampled at 3x3x3mm). Resulting normalization parameters were applied to functional images which were then subsequently smoothed using an 8-mm Gaussian kernel, full-width half-max. Finally, visual inspection was employed assessing EPI alignment to structural images after co-registration and accurate warping to the MNI standard space after normalization to assure quality of the preprocessing pipeline for images from all subjects and runs.

#### fMRI data analysis

General linear models were defined separately for each participant. Blocks were modeled as boxcar functions convolved with the canonical double-gamma hemodynamic response function (HRF). Six motion parameters were included as covariates of no interest, as were individual volumes representing global signal intensity change ≥2.5 standard deviations from average. Contrast images were created at the subject-level for the contrast of interest, Observe vs. Shape Match, which provided an index of trauma-related emotional reactivity. To investigate group-level differences in amygdala reactivity at baseline (i.e., PTSDs vs. TEHCs), the contrast images were entered in a random-effects analysis using a two sample t-test in the GLM Flex statistical software package with a search restricted to the amygdala, defined anatomically via the AAL atlas and thresholded at *p* < 0.005, 5 contiguous voxels (135 mm^3^), corresponding to a false-positive discovery rate of 5% as estimated by 10,000 Monte Carlo simulations using AlphaSim. Resulting significant activation clusters in the amygdala were used to define functional region(s) of interest (ROI) reflecting specific areas of amygdala hyperreactivity. Subsequently, averaged parameter estimates were extracted from these ROIs using MarsBaR (Brett et al., 2002) from both pre- and post-intervention fMRI data for Observe vs. Shape Match for the PTSDs only, and entered in a regression analysis in SPSS (thresholded at *p* < 0.05) to assess the relationship between neural changes and changes in PTSD symptoms following the intervention. One TEHC and three PTSDs were excluded from fMRI analyses due to fMRI technical issues and/or missing data.

#### Clinical outcomes data analysis

In computing the required sample size, we used a power level of 0.80, *α* = 0.05, *d* = 0.60, estimating a total sample size of 19 (Faul et al., 2007). The primary clinical variables of interest were changes in CAPS-5 and PCL-5 scores, as these reflect commonly-used and well-validated clinician- and self-reported PTSD symptom measures, respectively. We additionally examined potential changes in depression symptoms and positive and negative affect following the affect labeling intervention to examine more general effects. We used SPSS to conduct independent-samples t-tests for variables of interest to examine differences between PTSD and TEHC at baseline to validate group status, as well as linear mixed-model and paired t-test analyses of variables of interest to assess changes from pre to post intervention for participants with PTSD for intent-to-treat and completer-only analyses, respectively. In the linear mixed model, time (pre, post) was the independent variable and symptoms (e.g., CAPS, PCL) was the dependent variable. We examined the coefficient associated with time for statistical significance and this coefficient represented the predicted change in the dependent variable from pre to post intervention. The mixed model included all participants with available data for at least one timepoint, meaning that those with available data only at baseline (*N* = 7) were included in the analysis in addition to those with complete data (*N* = 13). We additionally examined possible differences between those with PTSD who completed vs. dropped out of the intervention by conducting independent-samples t-tests. All results of this pilot study were thresholded at *p* < 0.05 to establish proof-of-concept for our novel intervention. Cohen’s d effect sizes were also calculated.

## Results

### Baseline data comparing PTSD and TEHC

**Figure 3:**
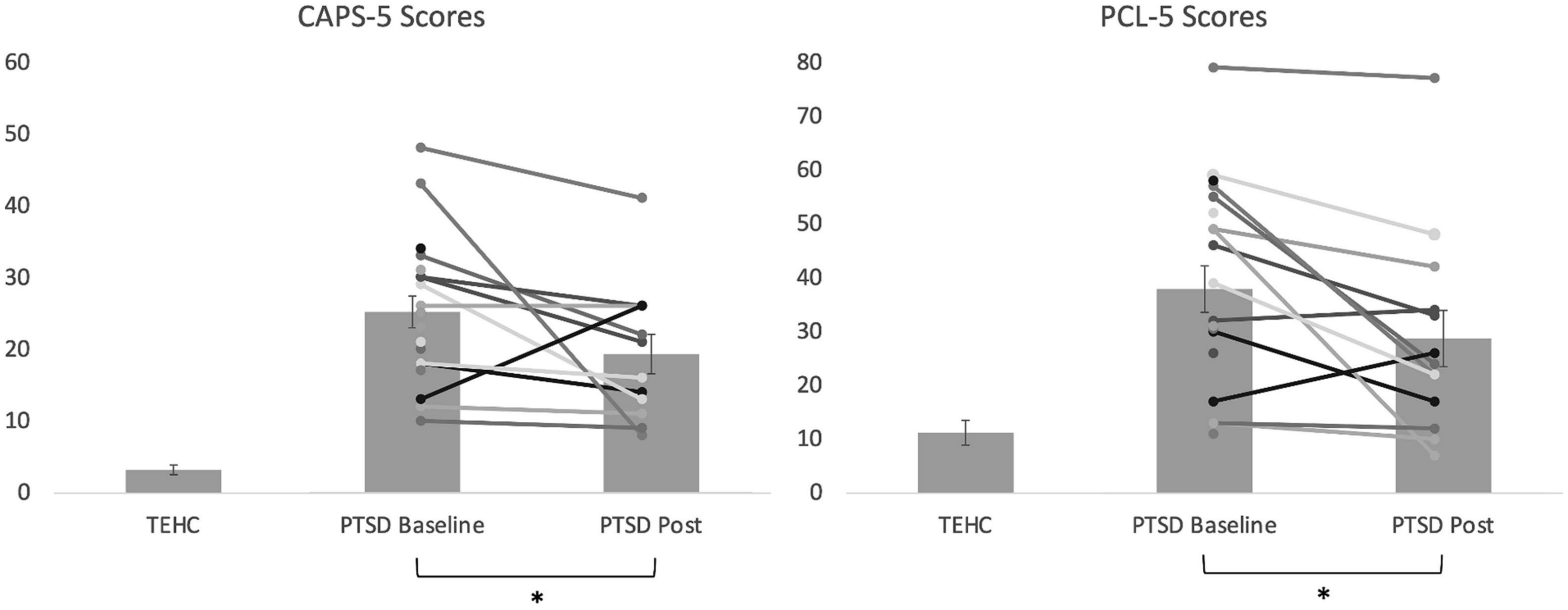
Bar graph illustrating average CAPS-5 and PCL-5 scores at baseline and post-intervention for the PTSD group, as well as at baseline for the TEHC group, with individual data points overlayed for the PTSD group. Higher scores reflect greater severity. Error bars reflect standard error of the mean for each group. The reduction from baseline to post was significant in the PTSD group for both CAPS-5 and PCL-5 scores (*p < 0.05) in intent-to-treat analyses, as also shown in Table 2.

**Figure 4:**
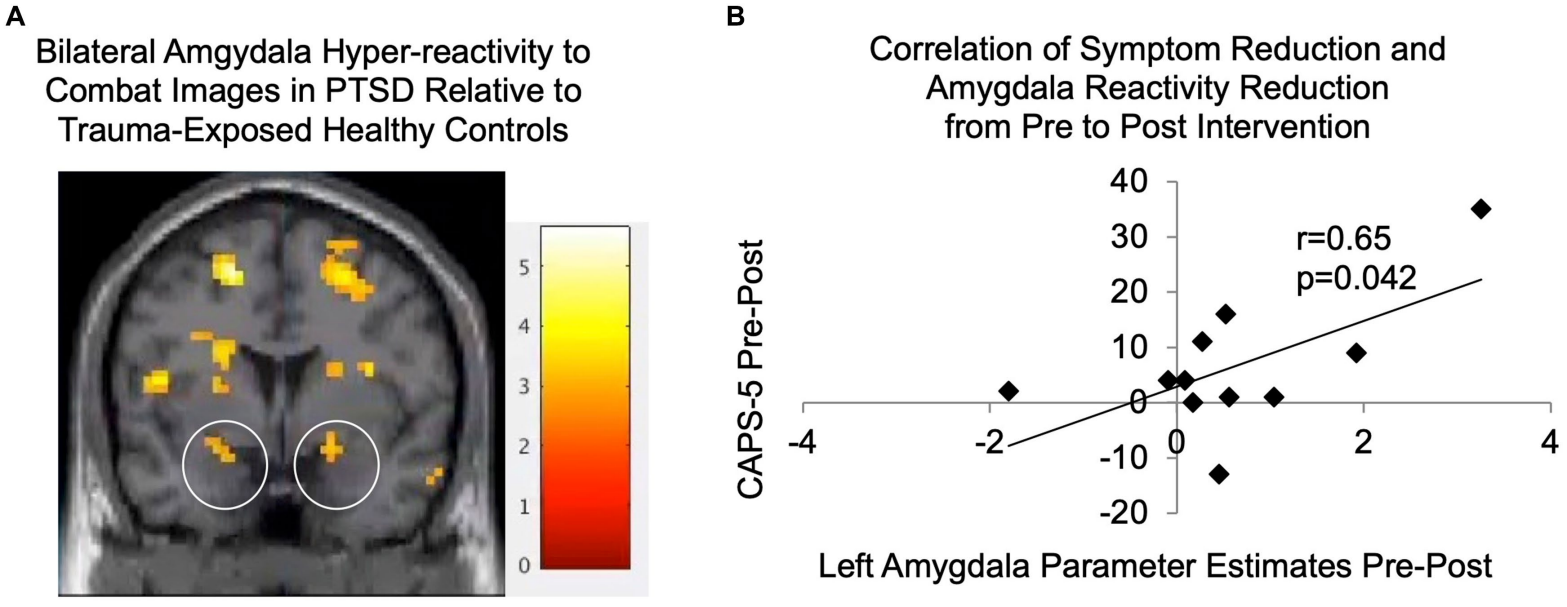
**(A)** Significantly greater activity was seen in bilateral amygdala (the activations located within the circled regions) in PTSDs (*N* = 17) relative to TEHCs (*N* = 19) during passive observation of combat scenes at baseline prior to the intervention (*p* < 0.005; shown at coronal slice *y* = 2 with gray scale of t-scores). **(B)** Parameter estimates were extracted for participants with PTSD from the functionally-defined left amygdala cluster, as well as from the corresponding region from post-intervention fMRI data; reductions in these left amygdala parameter estimates were significantly correlated with reductions in CAPS-5 scores from pre- to post-intervention (*N* = 11, *r* = 0.65, *p* = 0.042). CAPS-5, Clinician-Administered PTSD Scale for DSM-5.

Baseline demographic and clinical data (including mean and standard deviations, where appropriate) are shown in Table 1. As shown in Table 2, PTSD and TEHC participants did not significantly differ on any demographic variables including gender, age, years of active duty, ethnicity, employment status, student status, or education level. As expected, at baseline, the PTSD group had significantly higher levels of PTSD symptoms relative to TEHC, as measured by CSR, PCL-5, and CAPS-5 scores (see Table 2 and Figure 3). Also as expected, the PTSD group endorsed significantly higher depression and negative affect, as well as lower positive affect, relative to TEHC (see Table 2). The PTSD group also reported significantly higher levels of combat exposure than TEHC, consistent with prior work suggesting that increased combat exposure is a risk factor for PTSD (Smith et al., 2008; see Table 2). At the neural level, PTSD showed significantly greater bilateral amygdala reactivity to trauma-related stimuli than TEHC, also as expected (left amygdala MNI coordinates: −18, 2, −17, 8 voxels, t(34) = 2.96, *p* < 0.005; right amygdala MNI coordinates: 21, 2, −11, 15 voxels, t(34) = 3.21; *p* < 0.005; see Figure 4A).

**Table 1:** Demographic and clinical data.

	TEHC		PTSD All		PTSD Completers Only				PTSD Drops Only	
	Pre Mean (*n* = 20)	SD	Pre Mean (*n* = 20)	SD	Pre Mean (*n* = 13)	SD	Post Mean	SD	Pre Mean (*n* = 7)	SD
Gender M/F	16/4		19/1		12/1				7/0	
Age range	23–44		22–45		22–45				26–34	
Age	31.15	(5.21)	31.25	(6.16)	31.54	(7.47)			30.71	(2.81)
Yrs Active Duty	4.60	(2.24)	5.40	(3.05)	5.23	(3.53)			5.71	(2.06)
Ethnicity										
Caucasian	7		5		3				2	
African American	1		1		1				0	
Hispanic/Latino	6		11		6				5	
Asian	3		1		1				0	
Mixed	3		2		2				0	
Employment Status										
Full-time	8		3		2				1	
Part-time	3		8		5				3	
Unemployed/Looking	7		4		2				2	
Unemployed/Not Looking	2		5		4				1	
Student Status										
Full-time	11		10		8				2	
Part-time	2		2		1				1	
Not a student	7		7		4				3	
Highest Education Level										
High School	1		0		0				0	
Some College	10		17		11				6	
College Degree	6		0		0				0	
Some Grad	1		1		1				0	
Grad Degree	2		2		1				1	
PTSD-Related Diagnosis										
PTSD	0		11		8		2		3	
Other Trauma-Related Disorder^1^	0		9		5		5		4	
No Trauma Disorder					0		5			
Comorbid Disorders										
Major Depressive Disorder	0		5		4				1	
Persistent Depressive Disorder	0		1		0				1	
Generalized Anxiety Disorder	0		2		2				0	
Social Anxiety Disorder	0		2		2				0	
Adjustment Disorder	1		1		0				1	
Mild Alcohol Use Disorder	1		1		0				1	
CSR	0.10	(0.31)	5.20	(1.11)	5.23	(1.24)	3.92^2^	(1.83)	5.14	(0.90)
CAPS-5	3.25	(3.02)	25.30	(9.85)	25.62	(11.62)	19.42^2^	(9.52)	24.71	(6.08)
PCL-5	11.25	(10.31)	37.98	(19.29)	41.38	(19.83)	28.58^3^	(18.88)	31.65	(17.87)
MASQ – Anhedonic Depression	20.15	(5.37)	28.00	(7.31)	27.23	(8.12)	23.64^2^	(6.35)	29.43	(5.83)
Combat Exposure Scale	13.60	(9.47)	20.20	(8.91)	21.15	(10.33)	20.91^2^	(10.28)	18.43	(5.74)
PANAS – Positive	34.80	(6.59)	27.30	(10.06)	28.85	(9.90)	30.55^2^	(6.14)	24.43	(10.47)
PANAS – Negative	17.15	(5.52)	25.40	(9.86)	27.31	(9.81)	21.73^2^	(10.73)	21.86	(9.63)

^1^Participants who met criteria for other trauma-related disorder but not full PTSD met criteria for some but not all symptoms clusters, yet had clinically-significant distress or impairment that warranted treatment. ^2^Post-interview and questionnaire data is missing from one completer. ^3^One completer’s PCL-5 score was taken from the post fMRI session rather than one-month post interview to yield complete data (*n* = 13). SD, standard deviation; CSR, clinical severity rating; PCL-5, PTSD Symptom Checklist for DSM-5; CAPS-5, Clinician-Administered PTSD Scale for DSM-5.

**Table 2:** Statistics comparing demographic and clinical data.

	TEHC vs. PTSD	PTSD Completers vs. Drops	PTSD Pre vs. Post ITT	PTSD Pre vs. Post Completers Only
	Pre	Pre	Pre vs. Post	Pre vs. Post
Gender M/F	χ^2^(1) = 2.06, n.s.	χ^2^(1) = 0.57, n.s.		
Age	t(38) = −0.06, n.s.	t(16.82) = 0.35, n.s.^†^		
Years Active Duty	t(34.93) = −0.94, n.s.^†^	t(18) = −0.33, n.s.		
Ethnicity	χ^2^(4) = 3.00, n.s.	χ^2^(4) = 2.74, n.s.		
Employment Status	χ^2^(3) = 6.65, *p* = 0.084	χ^2^(3) = 0.92, n.s.		
Student Status	χ^2^(2) = 0.02, n.s.	χ^2^(2) = 1.35, n.s.		
Highest Education Level	χ^2^(4) = 8.82, *p* = 0.066	χ^2^(2) = 0.74, n.s.		
PTSD-Related Diagnosis		χ^2^(1) = 1.98, n.s.		
CSR	t(21.93) = −19.88, *p* < 0.001^†^**	t(18) = 0.17, n.s.	t(11.72) = 2.57, *p* = 0.025* d = 0.846	t(11) = 2.53, *p* = 0.028* d = 0.838
CAPS-5	t(22.55) = −9.57, *p* < 0.001^†^**	t(18) = 0.19, n.s.	t(14.70) = 2.16, *p* = 0.048* d = 0.607	t(11) = 1.94, *p* = 0.079 *d* = 0.584
PCL-5	t(29.04) = −5.47, *p* < 0.001^†^**	t(18) = 1.08, n.s.	t(13.29) = 2.82, *p* = 0.014* d = 0.493	t(12) = 2.96, *p* = 0.011* d = 0.661
MASQ – Anhedonic Depression	t(38) = −3.87, *p* < 0.001**	t(18) = −0.63, n.s.	t(12.98) = 2.47, *p* = 0.028*	t(10) = 1.76, n.s.
Combat Exposure Scale	t(38) = −2.27, *p* = 0.029*	t(18) = 0.64, n.s.	t(11.09) = 1.73, n.s.	t(10) = 1.84, *p* = 0.096
PANAS – Positive	t(32.78) = 2.79, *p* = 0.009^†^*	t(18) = 0.93, n.s.	t(14.28) = −1.02, n.s.	t(10) = −0.034, n.s.
PANAS – Negative	t(38) = −3.27, *p* = 0.002*	t(18) = 1.19, n.s.	t(10.22) = 2.08, *p* = 0.063	t(10) = 2.09, *p* = 0.063

^†^Degrees of freedom adjusted for unequal sample variances. ITT, intent-to-treat. *p < 0.05, **p < 0.001, with specific *p*-values indicated for *p* < 0.1. n.s. = *p* > 0.1.

### Baseline data comparing participants with PTSD who completed vs. dropped

Thirteen out of 20 participants with PTSD completed all six sessions of the affect labeling intervention, reflecting a 65% retention rate, which is similar to rates for CBT or PE (e.g., Eftekhari et al., 2013). It is noted, however, that one of these participants did not complete the post-fMRI session and another did not complete the final clinical interview. As shown in Table 2, participants with PTSD who completed the intervention (*N* = 13) did not differ significantly from those who dropped out of the study (*N* = 7) on any baseline demographic or clinical variables, suggesting no clear patterns or predictors for study dropout.

### Pre- to post-intervention changes

As shown in Table 2 and Figure 3, mixed model intent-to-treat analyses revealed significant reductions from pre- to post-intervention in both clinician-reported measures of PTSD symptoms (CSRs [t(11.72) = 2.57, *p* = 0.025, *d* = 0.846] and CAPS-5 [t(14.70) = 2.16, *p* = 0.048, *d* = 0.607]) and self-reported PTSD symptoms (PCL-5 [t(13.29) = 2.82, *p* = 0.014, *d* = 0.493]), with moderate to large effect sizes ranging from 0.49 to 0.85 depending on the measure. We also found significant reductions in depression symptoms and marginally significant reductions in negative affect. In analyses including completers only, results were generally similar for PTSD symptom measures, although significance dropped to marginal for the CAPS-5. Regarding individual intervention response, 83% of participants (10/12, noting that only 12 participants with PTSD had post-intervention clinical interview data) showed reduced symptoms (i.e., Pre-Post>0) on the CAPS-5, and 62% of participants (8/13) showed a reliable reduction in symptoms on the PCL-5 (>5 points).^1^ Additionally, 42% of participants (5/12) were in remission at study completion as they no longer had clinically-significant symptoms or impairment (i.e., CSR < 4). No adverse events (i.e., self-harm, suicide attempts, hospitalizations) were reported. Finally, treatment expectancy ratings completed after the second (*M* = 18.20, SD = 6.39) and final (*M* = 18.56, SD = 7.99) intervention sessions were not significantly correlated with symptom improvement as assessed with either the CAPS-5 (2nd session, *r* = 0.57, *p* = 0.11, final session, *r* = 0.46, *p* = 0.26) or PCL-5 (2nd session, *r* = 0.15, *p* = 0.67, final session, *r* = 0.38, *p* = 0.31), arguing against expectancy effects.

### Correlation of PTSD symptom and neural changes

Figure 4A shows significantly greater activity seen in PTSDs relative to TEHCs during passive observation of combat scenes prior to the intervention on a coronal slice at *y* = 2, including in bilateral amygdala (located within the two circles). As shown in Figure 4B, reductions in CAPS-5 scores were significantly correlated with reductions in left amygdala activation in response to trauma-relevant images from pre- to post-intervention (*r* = 0.65, *p* = 0.042). It is also noted that reductions in left amygdala activation in response to trauma-relevant images from pre- to post-intervention were not significantly different from zero (*p* = 0.078). Analogous analyses for the right amygdala were not significant.

## Discussion

This study provided preliminary evidence of the safety, feasibility, and efficacy of a novel, brief, affect labeling-based intervention for combat-related PTSD in U.S. veterans. We found significant improvements in terms of both independent clinician judgments of the severity and frequency of PTSD symptoms (i.e., CSR and CAPS-5 scores) as well as self-ratings of how much participants were bothered by these symptoms (i.e., PCL-5 scores). Of note, 42% of participants were in remission at study conclusion. At the neural level, we found evidence for amygdala hyperreactivity in response to trauma-related reminders prior to the intervention, consistent with prior fMRI research (Hayes et al., 2012). We then found that this amygdala hyperreactivity was reduced following the affect labeling intervention to the extent that PTSD symptoms were reduced, which suggests that symptom improvement was associated with meaningful changes in trauma-related processing in the brain.

These results, including symptom reduction, corresponding neural changes, and the rate of diagnostic change, are especially promising given the minimal nature of the intervention in terms of both patient and clinician time and ease of administration. Specifically, the affect labeling intervention comprised six one-hour sessions of automated delivery of computer-based stimuli with only minimal clinical supervision. This is substantially less session time than standard CBT, which often involves 12–16 weekly 60-90-min sessions. Importantly, this intervention was administered via computer, supervised by a research technician with only basic clinical training, which incurs much lower cost than many other treatment approaches requiring a more highly trained clinician.

While effect sizes were smaller than other more extended exposure-based treatments (e.g., *d* = 1.19 for intent-to-treat and *d* = 2.07 for completers of prolonged exposure for veterans of the wars in Iraq and Afghanistan; Tuerk et al., 2011), this intervention has important potential benefits that make it worthy of further investigation, in addition to the time and cost benefits mentioned above. Its computer-based format can facilitate dissemination and reach more individuals in general as it can be administered via any computer, tablet, or smart phone. Additionally, this type of treatment involving computer-based “cognitive training” may be more appealing to individuals otherwise disinclined to pursue mental health treatment and therefore reach an underserved segment of the patient population. Unlike many exposure-based interventions, our affect labeling intervention did not require disclosure or discussion of personal traumas with a therapist which may be a significant barrier for some. In fact, only 25% of the intervention training trials focused specifically on trauma-related stimuli, with the rest of the trials involving either other aversive stimuli or neutral stimuli.

During each session of our affect labeling intervention, participants viewed several trauma-related and other aversive images, thought about how they were feeling in response to each image, and then verbally/symbolically characterized their responses (e.g., by selecting “I feel anxious” from possible options on a computer screen). It is not entirely surprising that affect labeling can down-regulate emotional responses; indeed, affect labeling may represent a core process of the long-held folk wisdom that ‘talking about your feelings will make you feel better.’ However, in the absence of a comparison group of participants with PTSD randomized to complete an alternative intervention as a control, the precise role of affect labeling or other mediators of this intervention – above and beyond the effects of mere exposure to trauma-related images – remains unknown and thereby represents a limitation of this study. Affect labeling may facilitate increased engagement with the trauma stimuli (i.e., attention to and encoding of), thereby increasing meaningful exposure to feared stimuli (in the absence of the feared outcome) and facilitating extinction learning. Alternatively, affect labeling may yield effects entirely independent of extinction learning by engaging additional VLPFC-based inhibitory regulation processes and essentially increasing participants’ spontaneous use of affect labeling or VLPFC inhibitory regulation to down-regulate amygdala responses at either a conscious or subconscious level. This may then result in more effective management of anxiety symptoms or prevention of initial threat responses from escalating into excessive anxiety. Although we hypothesize that the affect labeling component constitutes an important augmentation to mere exposure, the unique contribution of affect labeling must be teased apart in future work. In any case, this intervention nonetheless has value, primarily in its brief, standardized, cost-effective approach to exposure therapy. Additional limitations of this study include the strict exclusion criteria (e.g., no more than mild TBI, exclusion for fMRI contraindications such as shrapnel injuries and metallic implants), small sample size, and potential impact of medication use which was reported by one healthy control participant and three with PTSD (including one who dropped and two completers).

We also note that the significance of the neural findings related to symptom reduction being exclusive to the left amygdala is not entirely clear. While bilateral amygdalae have been well-established as integral to fear and emotional processing (LeDoux, 1996), there are a number of theories regarding possible differential roles for the left vs. right amygdala. It has been hypothesized that left amygdala may be more involved with affective processing related to linguistic encoding, more elaborative processing, or more sustained processing, relative to right amygdala (Baas et al., 2004), or it may have differential temporal dynamics (Sergerie et al., 2008). On the other hand, one systematic review of fMRI studies found that the left amygdala was activated more often than right, regardless of stimulus type, task instructions, extent of elaborative processing, or differential habituation rates (Baas et al., 2004). Research on the functional lateralization of the amygdala in PTSD is limited, although differential connectivity for left vs. right amygdala with prefrontal regions in participants with PTSD has been noted (Bryant et al., 2008), suggesting that future exploration is warranted.

Overall, results from this proof-of-concept study are promising and suggest that affect labeling training as a treatment for PTSD warrants further investigation. In follow-up work, we plan to build upon this initial study by conducting a randomized controlled trial to address many of the limitations described above, further refine the intervention for a remote/web-based interface, examine effects of increasing the percentage of trauma-focused stimuli in the intervention, and pinpoint psychological and neural mediators in order to maximize effects. Potential benefits of this affect labeling intervention include significantly reduced overall patient and clinician time, a reduction in treatment costs, a less stigmatizing “cognitive” format, and a computer/web/app-based format that can reach many more individuals suffering with PTSD.

## Data availability statement

The original contributions presented in the study are included in the article/supplementary material, further inquiries can be directed to the corresponding author.

## Ethics statement

The studies involving humans were approved by the UCLA Institutional Review Board. The studies were conducted in accordance with the local legislation and institutional requirements. The participants provided their written informed consent to participate in this study.

## Author contributions

LJB: Conceptualization, Formal analysis, Funding acquisition, Investigation, Methodology, Project administration, Supervision, Validation, Writing – original draft, Writing – review & editing, Data curation, Visualization. CD: Data curation, Investigation, Methodology, Writing – review & editing, Software. AN: Data curation, Writing – review & editing, Investigation, Validation. JT: Data curation, Methodology, Software, Writing – review & editing, Investigation, Validation. LBr: Writing – review & editing, Investigation. MV: Writing – review & editing, Investigation. ML: Conceptualization, Formal analysis, Funding acquisition, Methodology, Supervision, Writing – review & editing, Project administration, Resources, Visualization. MC: Conceptualization, Formal analysis, Funding acquisition, Methodology, Project administration, Supervision, Writing – review & editing, Resources, Validation, Visualization.

## Footnotes

^1^ https://www.ptsd.va.gov/professional/assessment/adult-sr/ptsd-checklist.asp
